# Chrysin-Loaded Extracellular Vesicles Attenuate LPS-Induced Neuroinflammation in BV2 Microglial Cells In Vitro: A Novel Neuroprotective Strategy

**DOI:** 10.3390/molecules30153131

**Published:** 2025-07-25

**Authors:** Francesca Martina Filannino, Raffaella Soleti, Melania Ruggiero, Maria Ida de Stefano, Maria Antonietta Panaro, Dario Domenico Lofrumento, Teresa Trotta, Angela Bruna Maffione, Tarek Benameur, Antonia Cianciulli, Rosa Calvello, Federico Zoila, Chiara Porro

**Affiliations:** 1 Department of Clinical and Experimental Medicine, University of Foggia, I-71100 Foggia, Italy; francesca.filannino@unifg.it (F.M.F.); maria.destefano@unifg.it (M.I.d.S.); teresa.trotta@unifg.it (T.T.); angelabruna.maffione@unifg.it (A.B.M.); federico.zoila@unifg.it (F.Z.); 2 SFR ICAT, CRCI2NA, CHU Angers, CNRS, INSERM, Nantes Universite, University of Angers, F-49000 Angers, France; raffaella.soleti@univ-angers.fr; 3 Department of Biosciences, Biotechnologies and Environment, University of Bari, I-70125 Bari, Italy; melania.ruggiero@uniba.it (M.R.); mariaantonietta.panaro@uniba.it (M.A.P.); antonia.cianciulli@uniba.it (A.C.); rosa.calvello@uniba.it (R.C.); 4 Department of Biological and Environmental Sciences and Technologies, Section of Human Anatomy, University of Salento, I-73100 Lecce, Italy; dario.lofrumento@unisalento.it; 5 Department of Biomedical Sciences, College of Medicine, King Faisal University, Al-Ahsa 31982, Saudi Arabia

**Keywords:** neuroinflammation, extracellular vesicles, chrysin, microglia, anti-inflammatory, BV2 cells

## Abstract

Neuroinflammation, driven by activated microglia, contributes to the progression of neurodegenerative diseases. Extracellular vesicles mediate intercellular communication and influence immune responses. Chrysin, a natural flavone found in fruits and propolis, has demonstrated anti-inflammatory effects. This study explored the immunomodulatory potential of chrysin-loaded EVs (EVs-Chry) derived from BV2 microglial cells. BV2 cells were treated with chrysin for 24 h to assess cytotoxicity and proliferation. EVs were isolated from treated and untreated cells, characterized by nanoparticle tracking analysis, and applied to naïve BV2 cells prior to LPS stimulation. Effects on cell morphology, migration, cytokine expression (IL-1β, IL-6), inflammasome activity (caspase-1), and apoptosis-related protein Bcl-xL were investigated. Our results show that EVs-Chry significantly reduced LPS-induced cell proliferation, restored resting microglial morphology, and reduced migratory capacity. Furthermore, co-treatment with EVs-Chry and LPS reduced pro-inflammatory cytokines such as IL-1β, IL-6, and caspase-1 expression while enhancing anti-apoptotic Bcl-xL levels, indicating a shift toward an anti-inflammatory, neuroprotective micro-glial phenotype. Together, our results demonstrated that EVs-Chry have neuroprotective effects on LPS-induced microglial activation and modulate microglial responses to inflammatory stimuli, attenuating pro-inflammatory signaling and promoting cellular homeostasis. These findings support the therapeutic potential of EVs-Chry in the context of neuroinflammatory and neurodegenerative disorders.

## 1. Introduction

The microglia are cells of mesodermal origin that have been described as ramified, resident macrophages that populate the central nervous system (CNS) [1,2]. Microglia can be categorized based on their morphology and function, i.e., M0, M1, and M2 [3,4]. The M0 phenotype represents microglia in the resting state. These cells monitor the presence of pathogens in the local environment and sense the microenvironment by interacting with neurons, ependymal cells, and other glial cells, such as astrocytes [5]. As members of the mononuclear macrophage family, microglia also perform a range of functions similar to those of macrophages. These include the identification and monitoring of dead cells, pathogenic microorganisms, and endogenous or exogenous compounds [6,7]. The M1 (classically activated) phenotype is characterized by the production of pro-inflammatory cytokines (such as TNF-α, IL-6, and IL-1β), chemokines, and reactive oxygen species (ROS), which lead to an acute immune response. This phenotype also expresses the cell surface marker, CD86, which is associated with detrimental effects on functional nerve cells [8]. The M2 (alternatively activated) phenotype is characterized by the production of anti-inflammatory cytokines (e.g., IL-10) and tissue remodeling receptors (CD206, Arg-1, etc.), which promote tissue repair, debris removal, wound healing and restoration of brain homeostasis [9].

Neuroinflammation is a frequent occurrence in cases of neurodegenerative diseases, including Alzheimer’s and Parkinson’s diseases [10]. The process is driven by the polarization of glial cells, among which microglia have distinct activation profiles, including pro-inflammatory phenotypes. Specifically, microglial activation demonstrates a highly dynamic and often overlapping spectrum, with subsets of M2 activation resulting from a wide range of inflammatory mediators [11,12]. These different activation states allow microglia to respond by inducing or resolving inflammation, which can be deleterious or beneficial to neurons [10]. Following an infectious trigger or injury, pro-inflammatory signaling cascades are activated in microglia, including the nuclear factor kappa light chain enhancer of activated B cells (NF-κB) pathway. This activation leads to the production of pro-inflammatory cytokines such as tumour necrosis factor-α (TNF-α), interleukin-6 (IL-6), and interleukin-1β (IL-1β) [13]. These cytokines are released and spread to the nearby tissues, where they trigger a positive feedback loop that induces the activation of surrounding microglia and contributes to the sustained inflammation over time [14]. Several studies have used lipopolysaccharide (LPS) in vitro to stimulate neuroinflammation. LPS activates microglia, driving them towards a pro-inflammatory M1 phenotype characterized by the release of pro-inflammatory cytokines. The transcriptional responses triggered by LPS or IFN-γ have been analyzed, revealing upregulation of key M1 markers such as IL-1β, IL-6, TNF-α and NOS2 [3,6,9].

Extracellular vesicles (EVs) are biologically active, nanoscale, membrane-bound particles released by all cell types. They vary in size and are typically classified into three categories: small extracellular vesicles (sEVs) or mesh-sized EVs, medium-sized extracellular vesicles (mEVs) or microvesicles, and large extracellular vesicles (lEVs) or apoptotic bodies. Among these, sEVs and mEVs are the most extensively studied and are known to play key roles in cell-to-cell communication [15,16]. Indeed, studies have shown that under conditions of cellular stress, microglia were able to upregulate the release of EVs containing pro-inflammatory mediators, such as IL-1β, IL-6, TNF-α, and caspase-1, contributing to the propagation of neuroinflammation [17,18]. The ability of EVs to transport bioactive material between cells is crucial to their therapeutic potential. Due to the wide range of cargo they can carry, EVs display multifaceted functions, capable of exerting both neuroprotective and neurotoxic effects [19].

These EVs serve as vectors for targeted drug delivery and as potential biomarkers for conditions such as ischemia, inflammation, and neuropathy associated with cerebrovascular diseases. Given their dual role as producers and recipients of EVs, we believe that microglia is a potential therapeutic target in the orchestration of neuroinflammation and potentially in its resolution [20].

In this context, the identification of bioactive compounds capable of modulating microglial function is of growing interest. Among these, chrysin has attracted particular attention due to its promising therapeutic potential. Chrysin, chemically known as 5,7-dihydroxy-2-phenyl-4H-chromone-4-one or 5,7-dihydroxyflavone, is a flavonoid compound [21]. It is naturally found in various sources, including honey, propolis, *Oroxylum indicum* and *Passiflora caerulea*, and several mushroom species, such as *Lactarius deliciosus* [22,23].

Chrysin has demonstrated significant pharmacological activities in numerous studies, including antitumour effects against various cancers, such as breast and colorectal cancer. Research studies have highlighted its ability to modulate various molecular pathways, including NF-κB, STAT3, Notch1, PI3K and microRNA [24,25]. These properties contribute to the inhibition of different forms of tumour growth and metastasis. In addition, the potential of chrysin in regulating neuroinflammation is substantiated by its capacity to modulate pivotal molecular pathways, including NF-κB, PI3K/Akt/mTOR, and JNK signaling cascades, that are critically involved in inflammatory responses and cell survival [26,27,28]. Chrysin’s anti-inflammatory effects are linked to its ability to downregulate pro-inflammatory signaling pathways, leading to a reduction in the production of pro-inflammatory cytokines and the suppression of inflammatory enzymes expression such as inducible nitric oxide synthase (iNOS) and cyclooxygenase-2 (COX-2). Additionally, chrysin enhances the body’s antioxidant defenses through the activation of enzymes such as superoxide dismutase (SOD) and glutathione peroxidase [29,30,31].

The main limitation of chrysin is its poor bioavailability, mainly due to its high metabolism in the intestine, liver and several target cells, through conjugation, biotransformation and production of glucuronides and sulphate derivatives [32]. Chrysin shows a very low volume of distribution, and its oral bioavailability is approximately 0.003–0.02%. For this reason, it is crucial that chrysin is administered at concentrations up to 100 mg/kg to achieve the desired therapeutic levels [33]. However, it should be noted that this may entail a significant risk of toxicity as the lethal dose of oral chrysin is 4350 mg/kg [34,35].

Several studies have evaluated the strategy of using nanoparticles to facilitate the transport of chrysin across the blood–brain barrier (BBB). To enhance the efficiency of the chrysin passage across the BBB, we employed a novel strategy utilizing EVs derived from microglia [36]. This approach relied on the intrinsic targeting capabilities of EVs toward stem cells, together with their ability to transport functional RNAs and chrysin itself. This method also exploited the natural pathway to achieve more precise and efficient cargo delivery, compared to less targeted approaches using synthetic nanoparticle systems [37,38,39].

This study aimed to test the hypothesis that stimulation of BV2 microglial cells with chrysin promotes the release of EVs enriched with anti-inflammatory molecules, potentially contributing to the attenuation of inflammation within the brain parenchyma. Our findings provide valuable insights into the role of EVs in microglial communication, as well as their involvement in the diffusion and the delivery of anti-inflammatory agents in the brain. To evaluate the functional impact of these vesicles, EVs loaded with chrysin (EVs-Chry) were applied to resting BV2 microglial cells, followed by LPS stimulation. This approach was used to assess their capacity to induce microglial polarization towards an anti-inflammatory phenotype. We further investigated the responses of EVs-Chry-treated BV2 cells by analyzing morphological changes, cell proliferation, migration, and the expression and release of pro-inflammatory cytokines under controlled experimental conditions. Our results indicate that EVs play a significant role in communication between microglial cells and may serve as effective cargo transport systems that contribute to the regulation of brain neuroinflammation.

## 2. Results

### 2.1. Chrysin Cytotoxicity and Microglial Proliferation Effects

**Figure 1:**
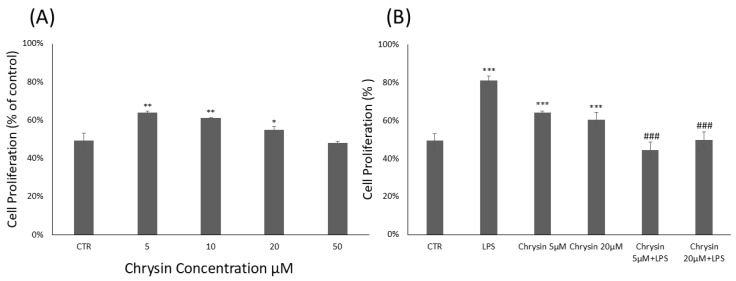
Chrysin effects on BV2 microglial cells proliferation. (**A**). Chrysin concentration-dependent effects on BV2 microglial cells. The results are representative of three independent experiments. (**B**) Effects of Chrysin at various concentrations on BV2 microglial cells proliferation in the absence or presence of LPS. Data are expressed as percentage of control values. Data are expressed as mean ± standard deviation (S.D.). * *p* < 0.05, ** *p* < 0.01, and *** *p* < 0.001 vs. control group; ### *p* < 0.001 vs. LPS-treated group.

To evaluate the cytotoxicity of chrysin (Figure 1A) and its effects on BV2 microglial cell viability, the CyQUANT^®^ Cell Proliferation Assay was performed. BV2 cells were treated with chrysin at concentrations ranging from 5 to 50 μM for 24 h. Our results showed that chrysin was non-cytotoxic at all tested concentrations. As shown in Figure 1A, treatment with chrysin at 5 μM, 10 μM and 20 μM significantly enhanced BV2 cell proliferation compared to untreated control cells. Based on these findings, the CyQUANT^®^ assay was repeated using 5 and 20 μM chrysin, with or without 1 μg/mL lipopolysaccharide (LPS), following a 24 h incubation (Figure 1B). Pre-treatment with chrysin in combination with LPS for 24 h significantly altered the viability of BV-2 microglial cells compared to the proliferative effects observed with LPS stimulation alone.

### 2.2. Isolation, Characterization and Cytotoxic Effect of Chrysin-Loaded EVs from Microglial Cells

**Figure 2:**
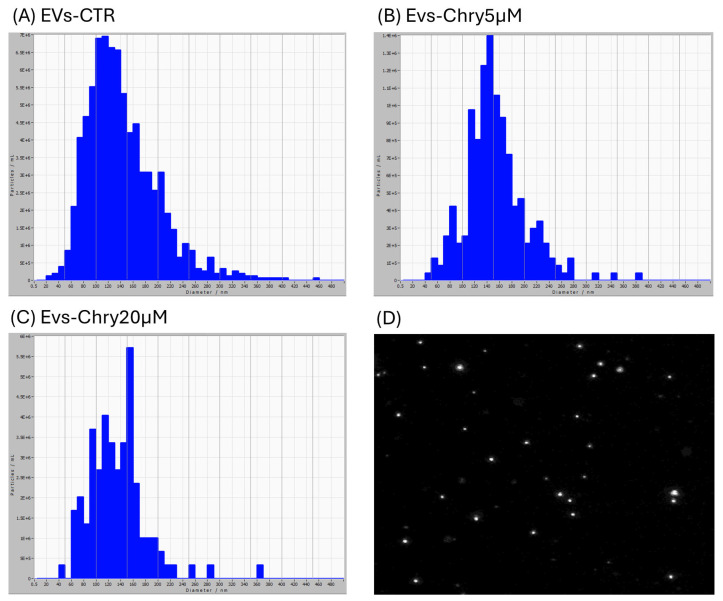
Characterization of EVs obtained from BV2 cell supernatant. EV samples examined with ZetaView are predominantly composed of vesicles with diameters ranging from 20 to 420 nm. In the EVs-CTR group (**A**), most vesicles show a diameter between 100 and 150 nm. The EVs-Chry5µM sample (**B**) shows a high number of vesicles with diameters ranging from 110 to 200 nm. The EVs-Chry20µM sample (**C**) presents slightly larger vesicles, with a diameter mainly ranging from 150 to 160 nm. Image of EVs obtained with ZetaView (**D**).

EVs were isolated from BV2 microglial cell lines under three conditions: untreated cells (control) and stimulated with chrysin at concentrations of 5 µM and 20 µM. After 24 h of treatment, EVs were isolated from the culture medium through a series of centrifugations, as described below. The isolated EVs—designated as EVs-CTR (from unstimulated cells; Figure 2A), EVs-Chry5µM (from cells treated with 5 µM chrysin; Figure 2B), and EVs-Chry20µM (from cells treated with 20 µM chrysin; Figure 2C) were initially analyzed for particle diameter distribution using the ZetaView nanoparticle tracking system.

Subsequently, EV concentrations were quantified using the BCA protein assay. These EVs were then applied to BV2 cells for an additional 24 h incubation to assess their biological activity.

**Figure 3:**
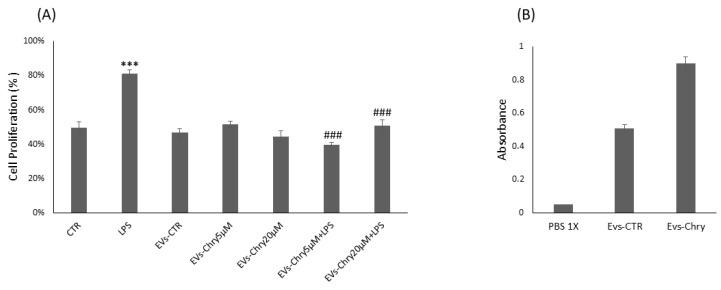
Effects of EVs-Chry5 µM and EV-Chry20 µM, at a concentration of 10 µg/mL, on the viability of BV2 microglial cells in the absence or presence of LPS (**A**). Cell proliferation was assessed using the CyQUANT^®^ assay. Absorbance of EVs at 348 nm (**B**). All results are representative of three independent experiments. Data are expressed as mean ± standard deviation (SD). *** *p* < 0.001 vs. control. ### *p* < 0.001 vs. LPS group.

Therefore, the CyQUANT^®^ assay was repeated after 24 h, with the addition of either EVs-Chry at 5 µM or at 20 µM, in the presence or absence of LPS. Pretreatment with EVs-Chry at 5 µM in combination with LPS had a significant effect on the viability of BV-2 microglial cells, resulting in the inhibition of LPS-induced proliferation (Figure 3A). Based on these results, EVs-Chry 5µM were selected for all subsequent analyses and are hereafter referred to as EVs-Chry. The absorbance at 348 nm (the λmax of chrysin) of the lysates from the EVs-CTR and EVs-Chry samples was analyzed. Figure 3B shows higher absorbance only in EVs-Chry. Further characterization of the vesicle preparation using the ZetaView system provided detailed information about the extracellular vesicles, including particle concentration and size distribution of the EV samples.

The vesicle samples were predominantly composed of vesicles ranging from 20 to 420 nm in diameter. In the EVs-CTR group, the majority of vesicles measured between 100 and 150 nm. The EVs-Chry5µM sample contained a high number of vesicles with diameters ranging from 110 to 200 nm. However, the EVs-Chry20µM sample displays slightly larger vesicles, with a diameter primarily ranging from 150 to 160 nm. Based on these size distributions, we concluded that EVs were successfully isolated from all samples, with a predominance of microvesicles and a relative reduction in exosome content.

### 2.3. BV-2 Cells Activated with LPS Show Anti-Inflammatory M2 State After EVs-Chry Stimulation

**Figure 4:**
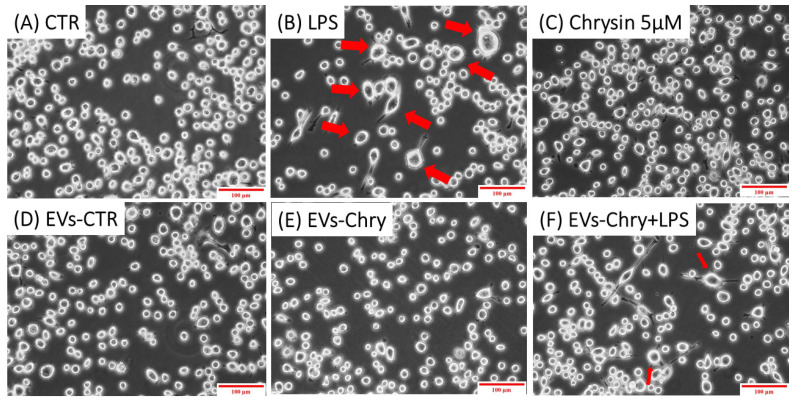
Effects of EV-chrysin on BV2 cell morphology over a 24-h period. Red arrows in the images highlight cells exhibiting morphological features characteristic of the M1 pro-inflammatory phenotype, including an enlarged cell body and retracted cellular processes. Control BV2 cells (**A**) and cells treated with EVs-CTR (**D**) show a quiescent microglial morphology. BV2 cells stimulated with LPS alone (**B**) show an amoeboid phenotype characteristic of the pro-inflammatory state, characterized by a small central body and numerous elongated processes. Treatment with EVs-Chry (**E**) and Chrysin (**C**) alone led to a ramified morphology characterized by a small central soma, very similar to the resting state observed in control cells both in size and shape. Cells treated with both EVs-Chry and LPS (**F**) showed a marked reduction in LPS-induced amoeboid morphology. Scale bar represents 100 µm (10× objective). Arrows in the images highlight cells exhibiting morphological changes. Cell area (µm^2^) was quantified using ImageJ software (version 1.8.0) with 64-bit Java 8 support.

The morphological changes observed in microglial cells are characterized by the progressive development of processes and cell body alterations in response to various stimuli. Therefore, the initial analysis focused on evaluating the morphological changes in BV2 microglial cells. Untreated control BV2 cells and BV2 cells treated with only EVs-CTR (Figure 4A), exhibited a quiescent microglial morphology, characterized by a small central body and numerous elongated processes. In contrast, BV2 cells stimulated with LPS only (Figure 4B) displayed an activated amoeboid shape, marked by an enlarged cell body and retracted processes, typical morphological characteristics of the M1 pro-inflammatory phenotype. As shown in Figure 4E, the treatment with EVs-Chry alone (Figure 4E) led to a ramified morphology characterized by a small central soma, closely resembling the resting state observed in control cells in both size and shape.

Cells treated with both EVs-Chry and LPS (Figure 4F) exhibited a marked reduction in the amoeboid morphology induced by LPS, suggesting that EVs-Chry exert anti-inflammatory effects and promote a shift toward the anti-inflammatory M2 phenotype.

**Figure 5:**
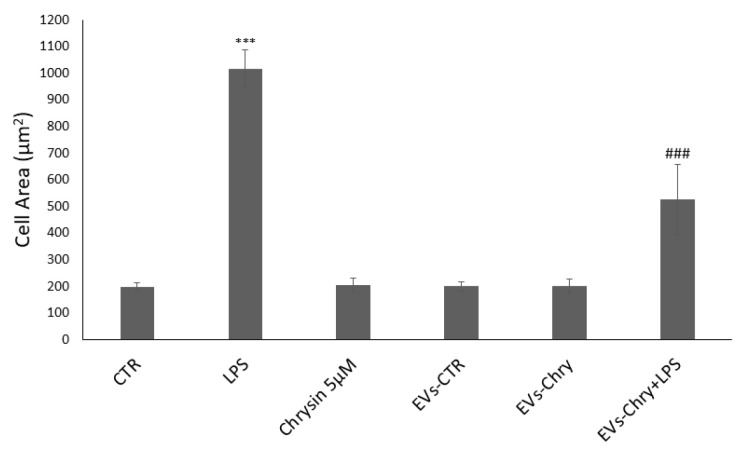
Quantitative analysis of cell areas. LPS-treated cells have a significant increase in cell size compared to controls. In contrast, cells treated with EVs-Chry and LPS showed a significant reduction in cell area, indicating a reversal of LPS-induced morphological alterations. Values are reported as mean ± standard deviation. A highly statistically significant difference was observed between the control and LPS-treated groups (*** *p* < 0.001), as well as between the EVs-Chry + LPS group and LPS-only groups (### *p* < 0.001).

The quantitative analysis of cellular areas (Figure 5) confirmed these observations visible under the optical microscope: LPS-treated cells showed a significant increase in cell size compared to controls. Conversely, cells treated with both EVs-Chry and LPS demonstrated a significant reduction in cell area, indicating a reversal of the LPS-induced morphological changes.

### 2.4. EVs-Chry Attenuate LPS-Induced Migration of BV2 Microglial Cells

**Figure 6:**
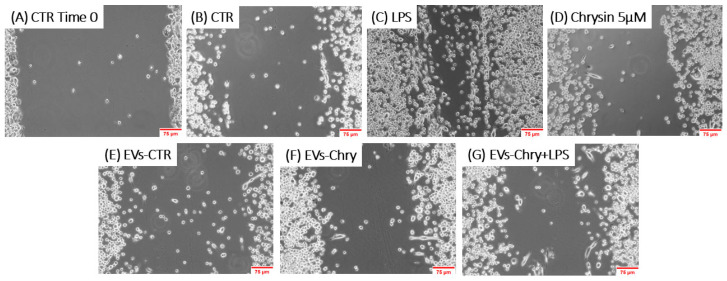
The analysis of the migratory capacity of microglia after the administration of EVs-Chry in the presence or absence of LPS. A wound was introduced into a sub-confluent monolayer of BV2 cells, and images of the wound area were captured at 0 and 24 h post-treatment: (**A**) BV2 cells at time 0, (**B**) 24 h under control conditions, (**C**) treated with LPS, (**D**) with Chrysin at 5 µM, (**E**) with EVs-CTR, (**F**) with EVs-Chry, and (**G**) cotreated EVs-Chry + LPS. The images shown are representative of one of three independent experiments. Scale bar: 75 µm (20× objective).

**Figure 7:**
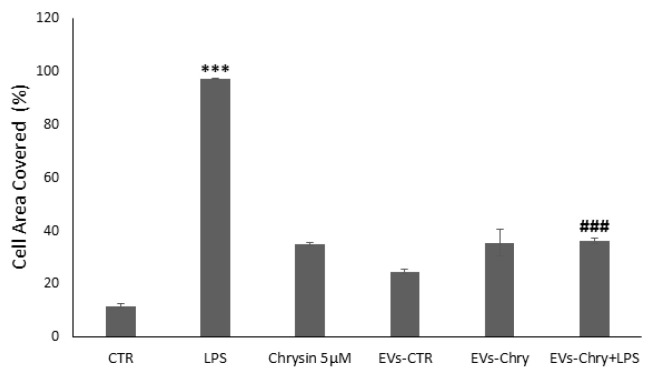
Wound closure was quantified using ImageJ software and subsequently plotted as the percentage of gap closure relative to the 0 h condition. Wound closure was quantified using ImageJ software, and results were plotted as the percentage of gap closure relative to the 0 h condition. Data are expressed as mean ± standard deviation. *** *p* < 0.001 vs. control; ### *p* < 0.001 vs. LPS-treated group.

To evaluate the effects of EVs-Chry on BV2 cells migration, a wound healing assay was performed. BV2 cells were scratched and treated with LPS, Chrysin (5 µM), EVs-CTR, EVs-Chry, or a combination of EVs-Chry and LPS. After a 24 h incubation period, BV2 cells treated with LPS alone (Figure 6C) exhibited a significantly accelerated rate of wound repair compared to the untreated cells (CTR). In contrast, treatment of BV2 with EVs-Chry (Figure 6F) resulted in wound opening comparable to that observed in CTR and EVs-CTR treated cells. Finally, co-treatment with EVs-Chry and LPS (Figure 6G) significantly reduced wound repair capacity compared to treatment with LPS alone, indicating an inhibitory effect on LPS-induced microglial migration. These findings are quantified in Figure 7, where analysis of the wound area confirms the statistical significance of both LPS and EVs-Chry+LPS treatments. Collectively, these results demonstrate that EVs-Chry attenuates BV2 microglial migration and may counteract the pro-migratory effects induced by LPS.

### 2.5. Suppression Effect of EVs-Chry on Expression of Pro-Inflammatory Molecules in BV2 Cells

In neurodegenerative diseases, inflammation plays a fundamental role in promoting both cell proliferation and migration. Indeed, prolonged inflammation can increase the levels of pro-inflammatory cytokines and proteases in the CNS, which can induce neuronal damage and cell death. In order to assess the anti-inflammatory potential of EVs-Chry, we evaluated its efficacy in suppressing the expression of pro-inflammatory cytokines. BV2 microglial cells were treated for 24 h with one of the following conditions: LPS alone, 5 µM chrysin, EVs-CTR, EVs-Chry alone, or a combination of EVs-Chry and LPS. As expected, LPS treatment significantly increased the expression of pro-inflammatory cytokines, including IL-1β and IL-6. However, EVs-Chry treatment reduced the expression levels of IL-1β and IL-6.

**Figure 8:**
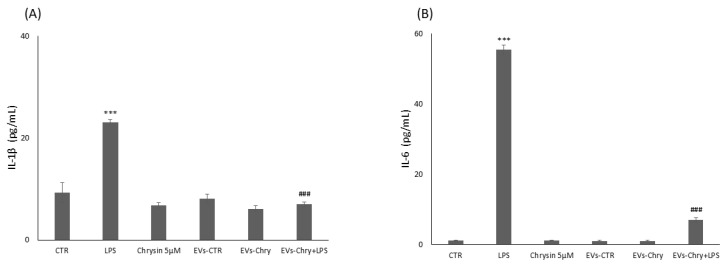
Modulatory effect of EVs-Chry on proinflammatory markers. Evaluation of IL-6 and IL1β expression is performed with ELISA following administration of EVs-Chry, with or without LPS. Data relating to cytokine concentrations of IL-6 (**A**) and IL-1β (**B**) show how EVs-Chry reduced the expression levels of IL-1β and IL-6. In particular, concomitant treatment with EVs-Chry and LPS significantly reduced the levels of these cytokines compared to treatment with LPS alone. Data are expressed as means (pg/mL) ± standard deviations (SD). (*** *p* < 0.001 compared to the control condition; and ### *p* < 0.001 compared to the condition of cells stimulated with LPS).

**Figure 9:**
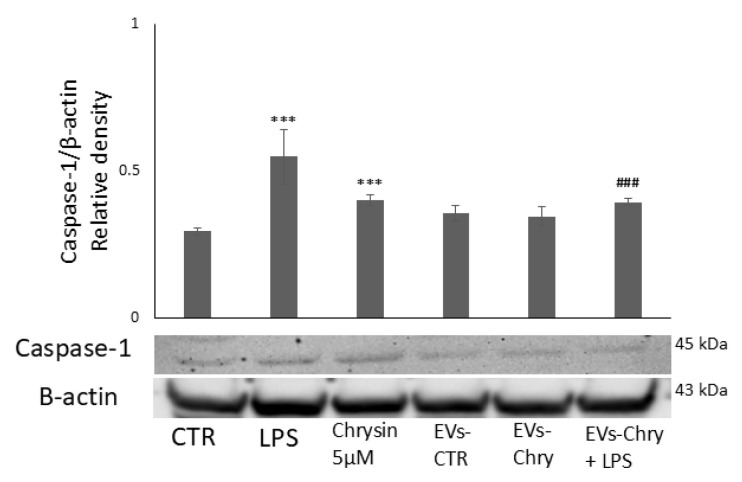
Modulatory effect of EVs-Chry on caspase-1. EVs-Chry modulate the expression of caspase-1, which is associated with the inflammasome pathway and appears increased in response to LPS stimulation, where it facilitates the release of IL-1β. Detection of caspase-1 expression by Western blotting and densitometric analysis. Data are expressed as mean ± standard deviation (SD). *** *p* < 0.001 vs. control. ### *p* < 0.001 vs. LPS group.

Furthermore, co-treatment with EVs-Chry and LPS significantly reduced the levels of these cytokines compared to LPS treatment alone (Figure 8). In addition, western blot analysis revealed a significant decrease in the protein expression level of caspase-1 following EVs-Chry treatment (Figure 9). Caspase-1 is associated with the inflammasome pathway and is upregulated in response to LPS stimulation, where it facilitates the release of IL-1β. These results suggest that EVs-Chry can drive microglial activation and promote the transition from pro-inflammatory state to neuroprotective phenotype by suppressing the expression of pro-inflammatory cytokines and associated inflammasome signaling components.

### 2.6. EVs-Chry Stimulate the Increase in the Anti-Apoptotic Protein Bcl-xL in BV-2 Cells

**Figure 10:**
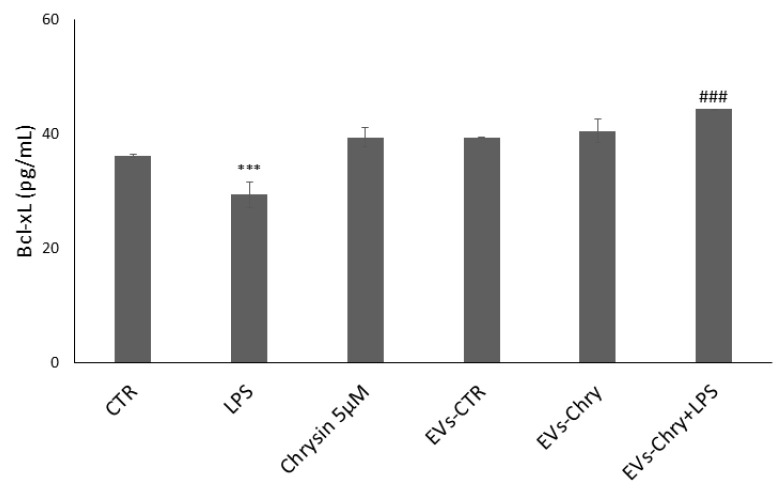
EVs-Chry prevents the downregulation of LPS-induced anti-apoptotic signaling and exerts a protective effect against inflammation-induced cell apoptosis. LPS treatment alone led to a significant reduction in Bcl-xL expression. In contrast, EVs-Chry treatment alone led to a marked upregulation of Bcl-xL expression compared to control (CTR), indicating a potential protective effect. Co-treatment with EVs-Chry in the presence of LPS effectively restored Bcl-xL expression. Assessment of anti-apoptotic Bcl-xL protein by ELISA. Data are expressed as means (pg/mL) ± standard deviations (SD). *** *p* < 0.001 compared to control condition. ### *p* < 0.001 compared to LPS-stimulated microglia condition. *** *p* < 0.001 compared to CTR.

To evaluate the impact of EVs-Chry on the regulation of the synthesis of mitochondria-associated apoptotic proteins, we analyzed the expression levels of the anti-apoptotic protein Bcl-xL (Figure 10). Treatment with LPS alone led to a significant reduction in Bcl-xL expression, consistent with the pro-apoptotic environment induced by inflammatory stimuli. In contrast, treatment with EVs-Chry alone resulted in a marked upregulation of Bcl-xL expression compared to the control (CTR), indicating a potential protective effect. In the course of our research, we examined the impact of a double EVs-Chry treatment in the presence of LPS. Furthermore, co-treatment with EVs-Chry in the presence of LPS effectively restored Bcl-xL expression to levels significantly higher than those observed with LPS treatment alone. These results suggest that EVs-Chry prevent LPS-induced downregulation of anti-apoptotic signaling and exert a protective effect against inflammation-induced cellular apoptosis. This highlights the potential of EVs-Chry not only as an anti-inflammatory agent but also as a modulator of apoptosis through mitochondrial pathways.

## 3. Discussion

This study investigated the immunomodulatory potential of BV2-derived EVs-Chry. Chrysin promoted microglial proliferation without toxicity. Interestingly, our results show that EVs-Chry exhibit neuroprotective effects in the context of LPS-induced microglial activation. Indeed, EVs-Chry significantly reduced LPS-induced cell proliferation and restored resting microglial morphology while reducing migratory capacity. The EVs were able to modulate the responses of microglia to inflammatory stimuli, thereby attenuating pro-inflammatory signalling and promoting cellular homeostasis. The results of combined treatment with EVs-Chry and LPS show reduced expression of pro-inflammatory cytokines such as IL-1β, IL-6 and caspase-1, while increasing anti-apoptotic Bcl-xL levels.

In recent years, the importance of small cell-derived extracellular vesicles as key mediators of intercellular communication has become increasingly apparent. The CNS comprises several cell types, each of which is capable of releasing EVs in both in vitro and in vivo settings, contributing to physiological and pathological conditions in the brain [40]. EVs act as signal carriers between adjacent cells within the CNS, playing essential roles under normal physiological conditions, such as development and synaptic neurotransmission, as well as in pathological and neurodegenerative conditions. Cells release heterogeneous populations of EVs that vary in size and secretion pathways [41,42]. Based on their origin and physical characteristics, EVs can be classified based on biogenesis and size into three main categories: exosomes, microvesicles (MV), and apoptotic bodies. Exosomes are small EVs of nanometer size, typically 30–100 nm in diameter [43].

They form as intraluminal vesicles within endosomal multivesicular bodies (MVBs), which are released from cells into the extracellular environment upon fusion of MVBs with the plasma membrane [43]. MVs, also known as ectosomes, typically range from 100 to 1000 nm in diameter and originated from the external budding of the plasma membrane and are subsequently released into the extracellular space after selective incorporation of proteins, nucleic acids and lipids. Apoptotic bodies are larger, with diameters ranging from 500 nm to 5 μm, and are released into the extracellular space during the process of apoptosis [44,45]. As demonstrated in previous studies, EVs have been shown to possess intrinsic properties that are associated with a variety of immune responses and the regulation of complex intracellular pathways [46,47].

This has led to an increased recognition of their potential utility in the therapeutic management of various pathologies, including neurodegenerative conditions. Therefore, the present study specifically investigated the effects of EVs loaded with chrysin, a compound previously demonstrated to possess strong anti-inflammatory properties on microglial cells [48,49,50].

To assess the potential cytotoxicity of the EV samples, BV2 microglial cells were treated with various concentrations of EVs, including those loaded with chrysin, for 24 h. Cell viability was measured by quantifying DNA content, allowing for a direct assessment of cell number without reliance on metabolic activity assays. No significant cytotoxic effects were observed in any treatment group compared to the untreated control.

However, in the case of the sample treated with EVs-Chry at 5 µM and EVs-Chry at 20 µM, the vesicles demonstrated the ability to reverse the proliferative effect due to the inflammation caused by LPS. These treatments maintained cell viability at levels significantly lower than those observed in the LPS group, more closely resembling control levels. In the context of neuroinflammation, microglia cells are activated in response to different microenvironmental stimuli, by polarizing into a distinct activated state. These states are categorized into two different phenotypes, M1 (pro-inflammatory) and M2 (anti-inflammatory), that have different morphological and biological characteristics [51,52,53].

The M1 microglial phenotype is characterized by the release of pro-inflammatory cytokines and chemokines, which, when produced persistently, can lead to chronic neuroinflammation and neuronal damage. In contrast, activation of the M2 microglial phenotype results in the upregulation of anti-inflammatory cytokines and neuroprotective molecules, which in turn promote tissue repair and maintenance [54,55,56]. An imbalance in microglial polarization favoring the presence of the M1 phenotype at the expense of M2 is largely recognized as a contributing factor in the pathogenesis of neurodegeneration. Therefore, modulating microglial activation toward a more balanced or M2-dominant state is critical for preserving central nervous system homeostasis and may represent a promising therapeutic strategy for preventing neurodegeneration [57,58].

In our study, LPS was used as a pro-inflammatory stimulus to induce the activated M1 phenotype in vitro and analyze the morphological changes of BV2. Morphological analysis following LPS treatment revealed that microglia adopted a characteristic pro-inflammatory M1 amoeboid morphology, typified by enlarged, thickened cell bodies and reduced cellular processes. Quantitative assessment of the cell area demonstrated a significant increase in cell area compared to the control group. For the combined treatment group with EVs-Chry+LPS, microglia were dynamically directed towards a branched, non-amoeboid morphology. This phenotypic transition was associated with enhanced neuroprotective effects and a significant reduction in pro-inflammatory features. These findings support existing evidence suggesting that promoting microglial polarisation toward the M2 phenotype may offer a promising therapeutic strategy for alleviating neuroinflammation-related disorders [59,60].

From a physiological perspective, microglia play a key role in the process of synaptic pruning and remodeling processes, which are essential to maintain an optimal neural circuitry and brain connectivity. Under pathological conditions characterized by increased neuroinflammation, the activated pro-inflammatory M1 phenotype migrates to the site of injury [61]. This directed migration is mediated by an increased release of inflammatory mediators, such as TNF-α, monocyte chemotactic protein-1 (MCP-1), and ROS, prominently expressed at sites of neuroinflammation [62,63]. Furthermore, previous studies have demonstrated that in inflammatory environments, such as those stimulated by LPS, EVs released from cells can exacerbate the inflammatory message by further activation of microglia [62]. This propagation of inflammation not only enhances microglial activation but also exerts detrimental effects on all other brain cells. Thus, EVs serve as critical mediators of cell-to-cell communication by carrying signals that alter the phenotype of recipient cells [64].

LPS treatment is well documented to induce microglial phagocytosis, increase cellular migration and induce neuroinflammatory responses [65,66]. In our study, we established that LPS significantly enhanced microglial mobility, resulting in accelerated wound repair compared to the CTR. In contrast, treatment with EVs-Chry attenuated the LPS-induced increase in BV-2 cell migration, suggesting that EVs-Chry could alleviate inflammation-induced microglial activation. Our investigation focused on the later stages of chronic neuroinflammation, during which microglia upregulate inflammatory mediators such as IL-6 and IL-1β. IL-6 is a key proinflammatory cytokine implicated in several neurological disorders, including stroke and epilepsy [67,68]. Its biological activity is mediated via binding to the IL-6 receptor (IL-6R) [69,70].

Given the demonstrated anti-inflammatory properties of EVs-Chry, we investigated their impact on two key proinflammatory cytokines: IL-6 and IL-1β. Our findings show a significant reduction in the levels of both cytokines IL-6 and IL-1β in the presence of EVs-Chry and LPS, confirming the downregulating and protective effect of these vesicles.

In parallel, it is well established that activation of the NLRP3 inflammasome induces the autocleavage and activation of pro-caspase-1, which in turn facilitates the proteolytic maturation of the pro-inflammatory cytokines IL-1β and IL-18 [71,72]. Furthermore, activated caspase-1 also cleaves gasdermin D (GSDMD) and releases its N-terminal domain, which is transferred to the cell membrane and forms pores, mediating the release of cellular contents, including the inflammatory cytokines IL-1β and IL-18, and inducing the inflammatory cell death known as pyroptosis [73,74].

To evaluate the ability of EVs-Chry to modulate additional inflammatory pathways, we have measured the intracellular levels of caspase-1, an essential effector protease involved in inflammasome activation. As previously demonstrated, treatment with EVs-Chry resulted in a significant reduction in caspase-1 levels, even when induced by LPS stimulation [75,76].

Subsequently, we investigated the antiapoptotic effects of the released EVs-Chry by analyzing the expression of Bcl-xL, a member of the Bcl-2 family [77]. The Bcl-2 family plays a central role in regulating mitochondrial-mediated apoptosis, primarily through modulation of cytochrome c released from the mitochondrial intermembrane space, which initiates caspase activation and the execution phase of programmed cell death [78,79].

Co-treatment with EVs-Chry led to an upregulation of Bcl-xL expression even in the presence of LPS, thus suggesting that EVs-Chry may influence the activity of Bcl-2 family effectors and regulate apoptotic responses. These findings indicate that EVs-Chry enhance the expression of Bcl-xL, a physiological molecule that plays a pivotal role in counteracting LPS-induced toxicity in microglia. Importantly, EVs-CTR exhibited no comparable anti-inflammatory or anti-apoptotic activity in any of the experimental conditions tested. This observation is consistent with previous results [62] and underscores the critical importance of vesicle cargo composition in determining the functional properties of EVs, with EVs-Chry carrying a distinct bioactive load responsible for their observed therapeutic potential.

In conclusion, this study investigated the therapeutic potential of EVs-Chry as a strategy to enhance the cellular efficacy of chrysin delivery [80]. Here, we have assessed the anti-inflammatory effects of the isolated EVs-Chry and their possible neuroprotective potential, attenuating neuroinflammatory responses through a comprehensive set of in vitro analyses. A relevant aspect of our study is the result demonstrating the double anti-inflammatory and antiapoptotic effect played by EVs-Chry, indicating their potential to modulate the survival cellular pathways associated with neuroinflammation. Unlike the direct administration of free chrysin, the use of EVs as delivery vehicles offers several advantages due to their intrinsic biological composition. EVs naturally encapsulate a diverse cargo, including cellular proteins, DNA and RNA. EVs showed multiple benefits related to their biocompatibility, reduced immunogenicity, stability, pharmacokinetics, and biodistribution. Moreover, their efficient cellular uptake mechanisms further support their suitability as nanocarriers for therapeutic agents [81].

This use of EVs represents a particularly promising strategy for chrysin delivery, as EVs can cross the BBB and can selectively target specific cell types. They facilitate the transfer of both intravesicular cargo and membrane receptors to recipient cells [82]. These properties collectively and synergistically contribute to the observed anti-inflammatory and antiapoptotic effects, which closely mirror the direct effects of chrysin on cells. EVs offer a competitive advantage over synthetic drug delivery systems due to their low immunogenicity and cytotoxicity. Unlike EVs, synthetic lipid nanoparticles often elicit toxic immune responses in vivo and tend to accumulate mainly in the liver, limiting their effectiveness and raising safety concerns. EVs, due to their endogenous origin and high biocompatibility, are more likely to evade immune detection and clearance. However, despite their potential, the biogenesis, transport mechanisms, and cellular uptake of EVs remain poorly understood. Moreover, the complex biochemical nature of EVs raises several challenges, including the standard methods of isolation and purification and the efficiency of loading of substances. Therefore, further studies are necessary to fully characterize EVs and optimize their loading efficiency to prevent potential side effects and enhance their clinical application.

## 4. Materials and Methods

### 4.1. Microglial Cell Culture

In this study, the BV2 murine microglial cell line (American Type Culture Collection, Manassas, VA, USA) were maintained in Dulbecco’s Modified Eagle Medium (DMEM, Euroclone) supplemented with 10% fetal bovine serum (FBS; Euroclone, Milan, Italy), 100 U/mL penicillin, 100 μg/mL streptomycin, (penicillin–streptomycin; Euroclone), and 2 mM glutamine (glutamine; Euroclone)). Cultures were incubated at 37 °C in a humidified incubator with 5% CO_2_. Adherent BV2 cells were detached using Trypsin-EDTA (1X in PBS; Euroclone) and replated at the desired density. For the cell migration and morphological assays, cells were seeded into six-well plates. For viability assay, cells were seeded into 24-well plates and incubated for 24 h. Following incubation, they were divided into experimental groups. One group was treated with varying concentrations of chrysin, another was exposed to 1 μg/mL LPS from *Escherichia coli* O128: B12 (Sigma-Aldrich, St. Louis, MI, USA), and a third group was pretreated with chrysin for 2 h prior to LPS stimulation. After 24 h of treatment, cells were collected for further analysis.

### 4.2. EVs Production and Isolation

The murine BV2 microglial cell line was used for the production of extracellular vesicles (EVs). Cells were seeded and cultured to approximately 80% confluence as previously described, then treated with Chrysin at concentrations of 5 µM and 20 µM for 24 h. Following incubation, culture supernatants from both treated and untreated cells were collected and centrifuged at 1500× g for 10 min to remove cells and large debris. EVs were then isolated through successive centrifugation at 14,000× g for 45 min. The obtained EV pellets were washed and resuspended in 400 µL of (0.9% *w*/*v*) NaCl solution. The final supernatant from the washing step, which was free of EVs, served as the control (vehicle). EV concentration was determined by quantifying the total EV-associated proteins using the Qubit Protein Assay Kit with the Qubit^®^ 2.0 Fluorometer (Life Technologies, Eugene, OR, USA).

### 4.3. EVs Analysis

The isolated exosomes were characterized by nanoparticle tracking analysis (NTA) using the ZetaView system (Particle Metrix, Meerbusch, Germany). The particle size distribution and concentration were analyzed using the ZetaView instrument and its accompanying software. The system measures particle diameters within a range of 0–500 nm. Samples were diluted 1:1 with 1 × PBS to loading the measurement chamber. Data were collected from 11 distinct positions within the cell, with two measurement cycles performed for each sample. Nanoparticle tracking analysis (NTA) was conducted under standardized conditions: a temperature of 23 °C, sensitivity set to 85.0, a frame rate of 30 frames per second, and a shutter speed of 100. The total vesicle protein content was determined using the BCA Protein Assay Kit (Pierce, Rockford, IL, USA).

### 4.4. Crhysin Solution Preparation

Chrysin (Purity ≥ 99.4% (HPLC); MP Biomedicals) was initially dissolved in DMSO (dimethyl sulfoxide, cell culture reagent; MP Biomedicals) to prepare 1M stock solution. For experimental purposes, this stock solution of chrysin was diluted in DMEM to prepare the desired working concentrations. To assess the in vitro effects of chrysin, cells were pre-incubated with chrysin diluted in DMEM for up to 24 h.

### 4.5. Cell Proliferation Assay

The cytotoxic effects of chrysin on BV2 cells were evaluated using the CyQUANT^®^ Cell Proliferation Assay, a fluorescence-based method for quantifying cells and assessing cell proliferation and cytotoxicity. The BV2 cells were seeded in 96-well plates at a density of 5 × 10^4^ cells per well and incubated at 37 °C in a humidified atmosphere with 5% CO_2_ for 24 h. The initial chrysin concentrations used to assess cytotoxicity were 5 µM, 10 μM, 20 μM and 50 µM. Then the CyQUANT^®^ Assay was conducted using 5 µM and 20 μM of chrysin, both in the absence and presence of LPS, for 24 h. Fluorescence was measured using CLARIOstar^®^ Plus plate reader( BMG LABTECH SARL, Champigny s/Marne, France) with excitation/emission filters set to ~480 nm and ~520 nm, respectively.

### 4.6. Cell Morphology Analysis

Microglial morphology was analyzed by measuring the cell body area to determine the effect of chrysin on BV2 cells’ activation, both in the absence and presence of the pro-inflammatory stimulus LPS. Approximately 5 × 10^5^ cells were seeded into six-well plates. All morphological analyses were performed in triplicate. The results are presented as the mean cell body area, calculated from five independent experiments, with three cells analyzed per sample in each experiment. Morphological assessments were performed using a Leica DM IRB microscopy system (Leica Microsystems GmbH, Wetzlar, Germany) at 10× and 20× magnifications. Cell body areas (µm^2^) were quantified using ImageJ software version 1.8.0 with 64-bit Java 8 support.

### 4.7. Wound Healing Assay

To evaluate two-dimensional cell migration, a wound healing assay was performed using BV2 cells. A total of (1 × 10^6^) cells were seeded into six-well plates and cultured until reaching confluence. A uniform scratch was created in the cell monolayer using a sterile scraper. Following PBS washing and replacement with fresh DMEM, the remaining cells were incubated for 24 h under various conditions: chrysin, chrysin-loaded extracellular vesicles (EVs-Chry), or LPS treatment. All migration assays were conducted in triplicate. After 24 h, three representative images were captured per wound area for each condition using phase-contrast microscopy. Wound repair was quantified using ImageJ software and expressed as the percentage of the initial wound area covered by migrating cells, relative to the initial wound size at time zero (the start of the assay).

### 4.8. EISA Quantification of Cytokines

An ELISA test was performed on culture supernatants collected from the morphological assay. After 24 h of incubation, the culture medium was carefully aspirated and centrifuged at 1500 rpm for 10 min at 4 °C to remove residual cellular debris. The levels of the pro-inflammatory cytokines IL-6 and IL-1β, along with the anti-apoptotic protein Bcl-xL, were determined using an ELISA kit, following the manufacturer’s protocol (R&D system, a biotech brand; USA). Optical density (OD) was measured at 450 nm using a CLARIOstar^®^ Plus microplate reader (BMG Labtech, Ortenberg, Germany).

### 4.9. Protein Extraction and Western Blot Analysis

Following the treatment mentioned above, BV2 cells were gently detached from the culture plates by scraping and collected after centrifugation at 2000 rpm for 10 min at 4 °C. The obtained cell pellets were then lysed in an ice-cold lysis buffer containing 50 nM Tris-HCl (pH 8.0), 1% (*v*/*v*) Triton X-100, 1.5 M NaCl, 0.1% SDS, 1 μM leupeptin hemisulfate salt, 100 μM phenylmethylsulfonyl fluoride (PMSF), and 4 U/mL aprotinin (Sigma Aldrich). The cell lysates were subject to eight cycles of freezing and thawing, followed by centrifugation at 12,000 rpm for 30 min at 4 °C to remove insoluble debris. The supernatants were collected for further analysis. Western blotting was performed using primary antibodies against caspase-1 (1:500) and β-actin (1:500) (Santa Cruz Biotechnology, Inc., Heidelberg, Germany), with β-actin serving as a loading control to normalize protein expression levels. The membranes were incubated with the primary antibodies for 1 h at room temperature, followed by overnight incubation at 4 °C. Subsequently, membranes were incubated with horseradish peroxidase (HRP)-conjugated secondary antibodies (1:10,000); Santa Cruz Biotechnology) for 1 h at room temperature. Immunoreactive bands were visualized using a chemiluminescence detection system (BioRad Laboratories, Hercules, CA, USA). Band intensities were quantified by densitometric analysis using ImageJ software, and results are reported in arbitrary units.

### 4.10. Statistical Analysis

Statistical analysis was carried out using Statgraphics Centurion (Statgraphics Technologies Inc., The Plains, VA, USA). Comparisons between groups were conducted using two-sample tests, one-way analysis of variance (ANOVA), followed by Tukey’s post hoc test for multiple comparisons. Results from five independent experiments are expressed as mean ± standard deviation (SD), with all experiments conducted in triplicate. Statistical significance was set at *p*-value < 0.05.

## Data Availability

The original contributions presented in this study are included in the article. Further inquiries can be directed to the corresponding author(s).

## References

[1] Rauf A., Badoni H., Abu-Izneid T., Olatunde A., Rahman M., Painuli S., Semwal P., Wilairatana P., Mubarak M.S. (2022). Neuroinflammatory Markers: Key Indicators in the Pathology of Neurodegenerative Diseases. Molecules.

[2] Gong P., Jia H.-Y., Li R., Ma Z., Si M., Qian C., Zhu F.-Q., Sheng-Yong L. (2023). Downregulation of Nogo-B Ameliorates Cer-ebral Ischemia/Reperfusion Injury in Mice through Regulating Microglia Polarization via TLR4/NF-kappaB Pathway. Neurochem. Int..

[3] Wang M., Pan W., Xu Y., Zhang J., Wan J., Jiang H. (2022). Microglia-Mediated Neuroinflammation: A Potential Target for the Treatment of Cardiovascular Diseases. J. Inflamm. Res..

[4] Zhang Y., Miao L., Peng Q., Fan X., Song W., Yang B., Zhang P., Liu G., Liu J. (2022). Parthenolide modulates cerebral ischemia-induced microglial polarization and alleviates neuroinflammatory injury via the RhoA/ROCK pathway. Phytomedicine.

[5] 5Ajami B., Bennett J.L., Krieger C., Tetzlaff W., Rossi F.M.V. (2007). Local self-renewal can sustain CNS microglia maintenance and function throughout adult life. Nat. Neurosci..

[6] Dubbelaar M.L., Kracht L., Eggen B.J.L., Boddeke E.W.G.M. (2018). The Kaleidoscope of Microglial Phenotypes. Front. Immunol..

[7] Nimmerjahn A., Kirchhoff F., Helmchen F. (2005). Resting Microglial Cells Are Highly Dynamic Surveillants of Brain Parenchyma in Vivo. Science.

[8] Von Bernhardi R., Heredia F., Salgado N., Muñoz P. (2016). Microglia Function in the Normal Brain. Adv. Exp. Med. Biol..

[9] Cherry J.D., Olschowka J.A., O’Banion M.K. (2014). Neuroinflammation and M2 microglia: The good, the bad, and the inflamed. J. Neuroinflamm..

[10] Cronk J.C., Kipnis J. (2013). Microglia–the Brain’s Busy Bees. F1000Prime Rep..

[11] Spiers J.G., Vassileff N., Hill A.F. (2022). Neuroinflammatory Modulation of Extracellular Vesicle Biogenesis and Cargo Loading. NeuroMolecular Med..

[12] Bourgognon J.-M., Spiers J.G., Robinson S.W., Scheiblich H., Glynn P., Ortori C., Bradley S.J., Tobin A.B., Steinert J.R. (2021). Inhibition of neuroinflammatory nitric oxide signaling suppresses glycation and prevents neuronal dysfunction in mouse prion disease. Proc. Natl. Acad. Sci. USA.

[13] Liddelow S.A., Guttenplan K.A., Clarke L.E., Bennett F.C., Bohlen C.J., Schirmer L., Bennett M.L., Münch A.E., Chung W.-S., Peterson T.C. (2017). Neurotoxic reactive astrocytes are induced by activated microglia. Nature.

[14] Rőszer T. (2015). Understanding the Mysterious M2 Macrophage through Activation Markers and Effector Mechanisms. Mediat. Inflamm..

[15] Scalavino V., Liso M., Cavalcanti E., Gigante I., Lippolis A., Mastronardi M., Chieppa M., Serino G. (2020). miR-369-3p modulates inducible nitric oxide synthase and is involved in regulation of chronic inflammatory response. Sci. Rep..

[16] Vassileff N., Cheng L., Hill A.F. (2020). Extracellular vesicles–propagators of neuropathology and sources of potential biomarkers and therapeutics for neurodegenerative diseases. J. Cell Sci..

[17] Kumar A., Stoica B.A., Loane D.J., Yang M., Abulwerdi G., Khan N., Kumar A., Thom S.R., Faden A.I. (2017). Microglial-derived microparticles mediate neuroinflammation after traumatic brain injury. J. Neuroinflam..

[18] Brites D., Fernandes A. (2015). Neuroinflammation and Depression: Microglia Activation, Extracellular Microvesicles and mi-croRNA Dysregulation. Front. Cell Neurosci..

[19] D’EGidio F., Castelli V., D’ANgelo M., Ammannito F., Quintiliani M., Cimini A. (2024). Brain incoming call from glia during neuroinflammation: Roles of extracellular vesicles. Neurobiol. Dis..

[20] Yan B., Liao P., Liu Y., Han Z., Wang C., Chen F., Lei P. (2024). Therapeutic potential of microglia-derived extracellular vesicles in ischemic stroke. Int. Immunopharmacol..

[21] Wang A.-P., Tian Y., Zhang W., Tian T., Gong S.-X., Huang W.-Q., Zhou Q.-Y. (2021). Microglia-associated neuroinflammation is a potential therapeutic target for ischemic stroke. Neural Regen. Res..

[22] Théry C., Witwer K.W., Aikawa E., Alcaraz M.J., Anderson J.D., Andriantsitohaina R., Antoniou A., Arab T., Archer F., Atkin-Smith G.K. (2018). Minimal information for studies of extracellular vesicles 2018 (MISEV2018): A position statement of the International Society for Extracellular Vesicles and update of the MISEV2014 guidelines. J. Extracell. Vesicles.

[23] Talebi M., Talebi M., Farkhondeh T., Kopustinskiene D.M., Simal-Gandara J., Bernatoniene J., Samarghandian S. (2021). An Up-dated Review on the Versatile Role of Chrysin in Neurological Diseases: Chemistry, Pharmacology, and Drug Delivery Ap-proaches. Biomed. Pharmacother..

[24] Hadjmohammadi M.R., Nazari S.S.S.J. (2010). Separation optimization of quercetin, hesperetin and chrysin in honey by micellar liquid chromatography and experimental design. J. Sep. Sci..

[25] Talebi M., Talebi M., Farkhondeh T., Samarghandian S. (2020). Molecular Mechanism-Based Therapeutic Properties of Honey. Biomed. Pharmacother..

[26] Al-Hatamleh M.A.I., Boer J.C., Wilson K.L., Plebanski M., Mohamud R., Mustafa M.Z. (2020). Antioxidant-Based Medicinal Properties of Stingless Bee Products: Recent Progress and Future Directions. Biomolecules.

[27] Dinda B., SilSarma I., Dinda M., Rudrapaul P. (2015). *Oroxylum indicum* (L.) Kurz, an important Asian traditional medicine: From traditional uses to scientific data for its commercial exploitation. J. Ethnopharmacol..

[28] Kalogeropoulos N., Yanni A.E., Koutrotsios G., Aloupi M. (2013). Bioactive microconstituents and antioxidant properties of wild edible mushrooms from the island of Lesvos, Greece. Food Chem. Toxicol..

[29] Kamat S., Kumari M., Sajna K.V., Jayabaskaran C. (2020). Endophytic fungus, Chaetomium globosum, associated with marine green alga, a new source of Chrysin. Sci. Rep..

[30] Samarghandian S., Afshari J.T., Davoodi S. (2011). Chrysin reduces proliferation and induces apoptosis in the human prostate cancer cell line pc-3. Clinics.

[31] Moghadam E.R., Ang H.L., Asnaf S.E., Zabolian A., Saleki H., Yavari M., Esmaeili H., Zarrabi A., Ashrafizadeh M., Kumar A.P. (2020). Broad-Spectrum Preclinical Antitumor Activity of Chrysin: Current Trends and Future Perspectives. Biomolecules.

[32] Saleem S., Banerjee R., Kannan R.R. (2022). Chrysin-Loaded Chitosan Nanoparticle-Mediated Neuroprotection in Aβ_1–42_-Induced Neurodegenerative Conditions in Zebrafish. ACS Chem. Neurosci..

[33] Ibrahim S.S., Abo Elseoud O.G., Mohamedy M.H., Amer M.M., Mohamed Y.Y., Elmansy S.A., Kadry M.M., Attia A.A., Fanous R.A., Kamel M.S. (2021). Nose-to-Brain Delivery of Chrysin Transfersomal and Composite Vesicles in Doxorubi-cin-Induced Cognitive Impairment in Rats: Insights on Formulation, Oxidative Stress and TLR4/NF-kB/NLRP3 Pathways. Neuropharmacology.

[34] Xiao T., Pan M., Wang Y., Huang Y., Tsunoda M., Zhang Y., Wang R., Hu W., Yang H., Li L.-S. (2023). In vitro blood brain barrier permeability study of four main active ingredients from Alpiniae oxyphyllae fructus. J. Pharm. Biomed. Anal..

[35] Mishra A., Mishra P.S., Bandopadhyay R., Khurana N., Angelopoulou E., Paudel Y.N., Piperi C. (2021). Neuroprotective Poten-tial of Chrysin: Mechanistic Insights and Therapeutic Potential for Neurological Disorders. Molecules.

[36] Komath S., Garg A., Wahajuddin M., Din W. (2018). Development and evaluation of Chrysin-Phospholipid complex loaded solid lipid nanoparticles–storage stability and in vitro anti-cancer activity. J. Microencapsul..

[37] Li Q., Yang X., Li T. (2025). Natural flavonoids from herbs and nutraceuticals as ferroptosis inhibitors in central nervous system diseases: Current preclinical evidence and future perspectives. Front. Pharmacol..

[38] Xie Y., Xu Z., Wu C., Zhou C., Zhang X., Gu T., Yang J., Yang H., Zheng E., Xu Z. (2022). Extracellular vesicle-encapsulated miR-21-5p in seminal plasma prevents sperm capacitation via Vinculin inhibition. Theriogenology.

[39] Danis E.G., Mogulkoc R., Baltaci A.K. (2025). Flavonoids in Brain Ischemia-Reperfusion and their Effect on Kinases as Signaling Pathway Activity. CNS Neurol. Disord. Drug Targets.

[40] Gomes A.R., Sangani N.B., Fernandes T.G., Diogo M.M., Curfs L.M.G., Reutelingsperger C.P. (2020). Extracellular Vesicles in CNS Developmental Disorders. Int. J. Mol. Sci..

[41] Yang Z., Liu D., Zhou H., Tao B., Chang L., Liu H., Luo H., Wang D., Liu W. (2021). A New Nanomaterial Based on Extracellular Vesicles Containing Chrysin-Induced Cell Apoptosis Through Let-7a in Tongue Squamous Cell Carcinoma. Front. Bioeng. Biotechnol..

[42] Del Fabbro L., Bortolotto V.C., Ferreira L.M., Sari M.H.M., Furian A.F. (2025). Chrysin’s anti-inflammatory action in the central nervous system: A scoping review and an evidence-gap mapping of its mechanisms. Eur. J. Pharmacol..

[43] Zhang Q., Jeppesen D.K., Higginbotham J.N., Franklin J.L., Coffey R.J. (2023). Comprehensive isolation of extracellular vesicles and nanoparticles. Nat. Protoc..

[44] Deregibus M.C., Figliolini F., D’ANtico S., Manzini P.M., Pasquino C., De Lena M., Tetta C., Brizzi M.F., Camussi G. (2016). Charge-based precipitation of extracellular vesicles. Int. J. Mol. Med..

[45] Kalluri R., LeBleu V.S. (2020). The Biology, Function, and Biomedical Applications of Exosomes. Science.

[46] Mathieu M., Martin-Jaular L., Lavieu G., Théry C. (2019). Specificities of secretion and uptake of exosomes and other extracellular vesicles for cell-to-cell communication. Nat. Cell Biol..

[47] Marostica G., Gelibter S., Gironi M., Nigro A., Furlan R. (2021). Extracellular Vesicles in Neuroinflammation. Front. Cell Dev. Biol..

[48] Guo S., Wang H., Yin Y. (2022). Microglia Polarization From M1 to M2 in Neurodegenerative Diseases. Front. Aging Neurosci..

[49] Wang S., Chu C.-H., Stewart T., Ginghina C., Wang Y., Nie H., Guo M., Wilson B., Hong J.-S., Zhang J. (2015). α-Synuclein, a Chemoattractant, Directs Microglial Migration via H2O2-Dependent Lyn Phosphorylation. Proc. Natl. Acad. Sci. USA.

[50] Thawkar B.S., Kaur G. (2019). Inhibitors of NF-κB and P2X7/NLRP3/Caspase 1 Pathway in Microglia: Novel Therapeutic Opportu-nities in Neuroinflammation Induced Early-Stage Alzheimer’s Disease. J.Neuroimmunol..

[51] Rose-John S., Jenkins B.J., Garbers C., Moll J.M., Scheller J. (2023). Targeting IL-6 trans-signalling: Past, present and future prospects. Nat. Rev. Immunol..

[52] Kumari S., Dhapola R., Sharma P., Nagar P., Medhi B., HariKrishnaReddy D. (2024). The Impact of Cytokines in Neuroinflam-mation-Mediated Stroke. Cytokine Growth Factor Rev..

[53] Khan A.W., Farooq M., Hwang M.-J., Haseeb M., Choi S. (2023). Autoimmune Neuroinflammatory Diseases: Role of Interleukins. Int. J. Mol. Sci..

[54] Wang W., Wang Y., Wang F., Xie G., Liu S., Li Z., Wang P., Liu J., Lin L. (2024). Gastrodin regulates the TLR4/TRAF6/NF-κB pathway to reduce neuroinflammation and microglial activation in an AD model. Phytomedicine.

[55] Khaboushan A.S., Yazdanpanah N., Rezaei N. (2022). Neuroinflammation and Proinflammatory Cytokines in Epileptogenesis. Mol. Neurobiol..

[56] Pedersen S.S., Prause M., Williams K., Barrès R., Billestrup N. (2022). Butyrate Inhibits IL-1β-Induced Inflammatory Gene Ex-pression by Suppression of NF-κB Activity in Pancreatic Beta Cells. J. Biol. Chem..

[57] Huang Y., Xu W., Zhou R. (2021). NLRP3 inflammasome activation and cell death. Cell. Mol. Immunol..

[58] Bazan N.G., Marcheselli V.L., Cole–Edwards K. (2005). Brain Response to Injury and Neurodegeneration. Ann. N. Y. Acad. Sci..

[59] Okada S., Zhang H., Hatano M., Tokuhisa T. (1998). A Physiologic Role of Bcl-xL Induced in Activated Macrophages. J. Immunol..

[60] Filannino F.M., Panaro M.A., Benameur T., Pizzolorusso I., Porro C. (2024). Extracellular Vesicles in the Central Nervous System: A Novel Mechanism of Neuronal Cell Communication. Int. J. Mol. Sci..

[61] Guo W., Su L., Zhang H., Mi Z. (2022). Role of M2 macrophages-derived extracellular vesicles in IL-1β-stimulated chondrocyte proliferation and inflammatory responses. Cell Tissue Bank..

[62] La Torre M.E., Panaro M.A., Ruggiero M., Polito R., Cianciulli A., Filannino F.M., Lofrumento D.D., Antonucci L., Benameur T., Monda V. (2022). Extracellular Vesicles Cargo in Modulating Microglia Functional Responses. Biology.

[63] Chen Q., Che C., Yang S., Ding P., Si M., Yang G. (2022). Anti-inflammatory effects of extracellular vesicles from Morchella on LPS-stimulated RAW264.7 cells via the ROS-mediated p38 MAPK signaling pathway. Mol. Cell. Biochem..

[64] Abd Al Haleem E.N., Ahmed H.I., El-Naga R.N. (2023). Lycopene and Chrysin through Mitigation of Neuroinflammation and Oxidative Stress Exerted Antidepressant Effects in Clonidine-Induced Depression-like Behavior in Rats. J. Diet. Suppl..

[65] Xu M., Yang Y., Peng J., Zhang Y., Wu B., He B., Jia Y., Yan T. (2022). Effects of Alpinae Oxyphyllae Fructus on microglial polarization in a LPS-induced BV2 cells model of neuroinflammation via TREM2. J. Ethnopharmacol..

[66] Goyal A., Singh G., Verma A. (2023). A Comprehensive Review on Therapeutic Potential of Chrysin in Brain Related Disorders. CNS Neurol. Disord.–Drug Targets.

[67] Wang L., Zhao J., Zhu B., Shen J., Ye Z., Peng Q., Zhang Y. (2021). Microglia polarization in heat-induced early neural injury. Arch. Med Sci..

[68] Zhang N., Cui Y., Li Y., Mi Y. (2021). A Novel Role of Nogo Proteins: Regulating Macrophages in Inflammatory Disease. Cell. Mol. Neurobiol..

[69] Cheng L., Zheng M.-G., Jing J.-H., Yu S.-S., Li Z.-Y., Xu X.-Z., Yao F., Luo Y., Liu Y.-C. (2022). M1-type microglia can induce astrocytes to deposit chondroitin sulfate proteoglycan after spinal cord injury. Neural Regen. Res..

[70] Yao Y., Li J., Zhou Y., Wang S., Zhang Z., Jiang Q., Li K. (2023). Macrophage/microglia polarization for the treatment of diabetic retinopathy. Front. Endocrinol..

[71] Lee J.Y., Park C.S., Seo K.J., Kim I.Y., Han S., Youn I., Yune T.Y. (2023). IL-6/JAK2/STAT3 axis mediates neuropathic pain by regulating astrocyte and microglia activation after spinal cord injury. Exp. Neurol..

[72] Madry C., Kyrargyri V., Arancibia-Cárcamo I.L., Jolivet R., Kohsaka S., Bryan R.M., Attwell D. (2018). Microglial Ramification, Surveillance, and Interleukin-1β Release Are Regulated by the Two-Pore Domain K+ Channel THIK-1. Neuron.

[73] Lopez-Rodriguez A.B., Hennessy E., Murray C.L., Nazmi A., Delaney H.J., Healy D., Fagan S.G., Rooney M., Stewart E., Lewis A. (2021). Acute Systemic Inflammation Exacerbates Neuroinflammation in Alzheimer’s Disease: IL-1β Drives Ampli-fied Responses in Primed Astrocytes and Neuronal Network Dysfunction. Alzheimers Dement.

[74] Liang Z., Damianou A., Vendrell I., Jenkins E., Lassen F.H., Washer S.J., Grigoriou A., Liu G., Yi G., Lou H. (2024). Proximity proteomics reveals UCH-L1 as an essential regulator of NLRP3-mediated IL-1β production in human macrophages and microglia. Cell Rep..

[75] Luo L., Lin S., Hu G., Wu J., Hu Y., Nong F., Lu C., Chen R., Liu J. (2024). Molecular Mechanism of Rolupram Reducing Neuroinflammation in Noise Induced Tinnitus Mice through TLR4/NF kB/NLRP3 Protein/Caspase-1/IL-1 β Signaling Path-way. Int. J. Biol. Macromol..

[76] Liu X., Wang Y., Zeng Y., Wang D., Wen Y., Fan L., He Y., Zhang J., Sun W., Liu Y. (2023). Microglia-Neuron Interactions Promote Chronic Itch via the NLRP3-IL-1β-GRPR Axis. Allergy.

[77] Du G., Yang Z., Wen Y., Li X., Zhong W., Li Z., Zhang S., Luo E., Ding H., Li W. (2024). Heat stress induces IL-1β and IL-18 overproduction via ROS-activated NLRP3 inflammasome: Implication in neuroinflammation in mice with heat stroke. NeuroReport.

[78] Matsumoto S., Choudhury M.E., Takeda H., Sato A., Kihara N., Mikami K., Inoue A., Yano H., Watanabe H., Kumon Y. (2022). Microglial re-modeling contributes to recovery from ischemic injury of rat brain: A study using a cytokine mixture containing granulocyte-macrophage colony-stimulating factor and interleukin-3. Front. Neurosci..

[79] Liu Z., Wang F., Ma H., Xia H., Tian J., Sun T. (2020). Amentoflavone Induces Cell Cycle Arrest, Apoptosis, and Autophagy in BV-2 Cells. Front. Biosci. Landmark.

[80] Li Z., Chu S., He W., Zhang Z., Liu J., Cui L., Yan X., Li D., Chen N. (2019). A20 as a novel target for the anti-neuroinflammatory effect of chrysin via inhibition of NF-κB signaling pathway. Brain, Behav. Immun..

[81] Panaro M.A., Benameur T., Porro C. (2020). Extracellular Vesicles miRNA Cargo for Microglia Polarization in Traumatic Brain Injury. Biomolecules.

[82] Bang O.Y., Kim J.-E. (2022). Stem cell-derived extracellular vesicle therapy for acute brain insults and neurodegenerative diseases. BMB Rep..

